# Perovskite beyond solar: toward novel developments of lasers and detectors for photonic circuits

**DOI:** 10.1038/s41377-023-01197-0

**Published:** 2023-06-27

**Authors:** Alina Karabchevsky

**Affiliations:** School of Electrical and Computer Engineering, Ben-Gurion University of the Negev, Beer-Sheva, 8410501 Israel

**Keywords:** Optical materials and structures, Integrated optics

## Abstract

Possessing intriguing optoelectronic properties, metal halide perovskites can serve as a large-scale platform for miniaturized photonic circuits with on-chip active devices such as lasers and detectors.

## Abstract (web summary)

Metal halide perovskites have emerged as promising materials platform for integrated photonics applications. On chip nano-lasers and detectors owing to their ultracompact physical sizes, highly localized coherent output, and efficient waveguiding, are promising building blocks for fully integrated nanoscale photonic and optoelectronic devices.

## Graphical abstract

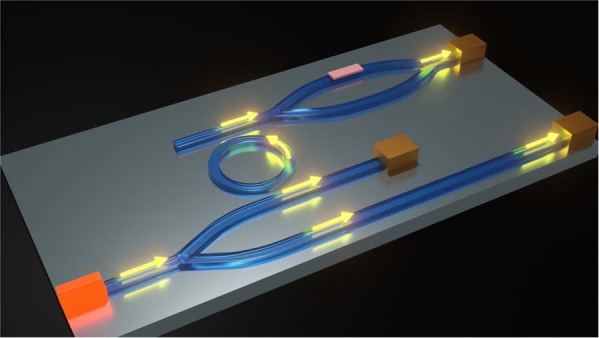

Over the past decade, one-dimensional (1D) micro or nanostructures based on organic/inorganic semiconductors have been demonstrated as an effective interconnects and as building blocks of miniaturized photonic circuits consisting two or more waveguides - a spatially inhomogeneous structures for guiding light. Due to their small dimensions, waveguides allow miniaturization and the design of efficient optical components on a chip^1,2^.

Hybrid organic–inorganic perovskites have emerged as new photovoltaic materials with impressively high-power conversion efficiency due to their high optical absorption coefficient and long charge carrier diffusion length. PIC can harness their optical properties enabling novel functionalities between Perovskite confined excitons and optical waveguide modes^3^. High-quality perovskite microwires can serve as an effective building block in micro- and nano-scale photonic circuits^4^. The integration of efficient light sources and detectors on a chip are highly desired. However, materials of photonic integrated circuitry dictate the functionality of the circuit. Today’s state-of-the-art fabrication, and functionalities, gives a snapshot of on-chip complexity currently achievable with perovskite and sub-group of perovskites waveguides as summarized in Table 1.

**Table 1:** Waveguides out of Perovskites

Perovskite type	Waveguide type	Architecture, Wavelengths of Operation	Ref.
Polycrystalline organic–inorganic perovskite	Electro-optical modulator	MAPbI_3_, MAPbBr_3_, MAPbBrxI_3−x_ and composition-graded perovskite MWs waveguide emission at ~535 nm, 605 nm and 770 nm, respectively.	^4^
Cesium Lead Halide Perovskites (CsPbX_3_, X = Cl, Br, I)	PL waveguiding and the photodetectors	single-crystalline horizontal CsPbX_3_ NWs and MWs with controlled orientation, excited 421 nm, PL emission peak at 528 nm	^6^
One-dimensional arrays of halide perovskite nanowires	Photodetectors	Surface-Guided CsPbBr_3_ NWs, PL ~ 520 nm	^7^
Methylammonium lead tri-iodide (MAPbI_3_)	Laser	Si3N4 waveguide integrated with MaPbl_3_ ring laser emission at ~790 nm.	^8^
Cesium lead halide perovskite	Laser	CsPbX_3_ (X = Cl, Br, I) NWs waveguides ~527 nm–534 nm	^9^
Lead halide perovskite	Laser	NWs waveguide emission ~500–800 nm of mixed lead halide perovskites	^10^
Hybrid halide perovskites	Amplifiers	Silicon substrate and cladded by a poly(methyl methacrylate) (PMMA) polymer waveguide, ASE@ 750 nm	^11,12^
Organic metal hybrid perovskitoid (OMHPs)	anisotropic optical waveguides, optical logic gate	Emission peak@428 nm, 476 nm, 524 nm	^13^
MAPbI_3_ perovskite	Photodetector	substrate: MAPbI_3_, FASn0.25Pb0.75I_3_, CsSn0.5Pb0.5I_3_, and FASn0.5Pb0.5I_3_), guiding layer: FTO, 750–1000 nm	^14^

*NWs* nanowires, *MWs* microwires, *PL* photoluminescence, *ASE* amplification of spontaneous emission

The attempt to create waveguides was shown for small areas perovskites and sub-group of perovskites. In Xu et al.^5^, scientists reported the method for controlled fabrication of areas of large-scale metal halide perovskite. The successful realization of crystallization method for the homogeneous perovskite single crystal arrays spanning 100 square centimeter areas. This method enables precise control over the crystal arrays, including different array shapes and resolutions with less than 10%-pixel position variation, tunable pixel dimensions from 2 μm to 8 μm as well as the in-plane rotation of each pixel. This may allow for a new type of photonic circuits with novel optoelectronics capabilities (Fig. 1).

**Fig. 1:**
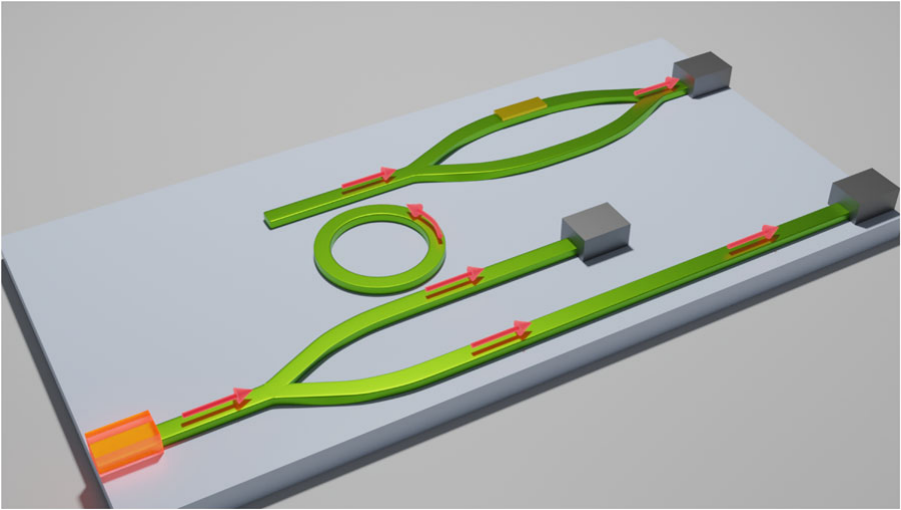
Schematic view of the photonic integrated circuit with active waveguides made of perovskite
